# Características Clínicas e Manejo de Pacientes Avaliados por Teleconsulta Cardiológica na Região Brasileira com Maior Número de Cidades Isoladas

**DOI:** 10.36660/abc.20220467

**Published:** 2023-04-27

**Authors:** Tarso Augusto Duenhas Accorsi, Renato Paladino Nemoto, Jairo Tavares Nunes, Antônio Fernando Barros de Azevedo, Flavio Tocci Moreira, Karen Francine Kohler, Karine de Amicis Lima, Bruna Dayanne Reges Amaral, Renata Albaladejo Morbeck, Carlos Henrique Sartorato Pedrotti

**Affiliations:** 1 Hospital Israelita Albert Einstein São Paulo SP Brasil Hospital Israelita Albert Einstein – Telemedicine, São Paulo, SP – Brasil

**Keywords:** Telecardiologia, Consulta Remota, Telemedicina, Encaminhamento e Consulta, População Suburbana

## Abstract

**Fundamento:**

As doenças cardiovasculares são a principal causa de morte no mundo. Regiões brasileiras geograficamente remotas e de baixa renda carecem de consultas especializadas. Não se tem conhecimento total acerca do manejo por telemedicina dessa população por parte de cardiologistas.

**Objetivos:**

Analisar a teleconsulta cardiológica na região brasileira com maior número de municípios isolados.

**Métodos:**

Entre fevereiro de 2020 e outubro de 2021, pacientes da Região Norte do Brasil avaliados por médicos generalistas locais foram encaminhados para avaliação cardiológica por telemedicina. Foram analisados os motivos do encaminhamento, dados demográficos, histórico clínico, exames físicos, exames complementares, medicamentos e prescrições pré e pós-telemedicina (considerou-se p<0,05 como estatisticamente significativo).

**Resultados:**

Analisamos 653 pacientes. A taxa de frequência foi de 85,7% (53,1% do sexo feminino, idade média: 54,2±6,5 anos). Os principais motivos de encaminhamento foram sintomas cardiovasculares (58,1%) e fatores de risco entre pacientes assintomáticos (13,3%). Apenas 12,6% apresentava alguma doença diagnosticada. A maioria dos pacientes havia passado por exame físico e eletrocardiogramas regulares. Poucos tinham exames complementares recentes. A prescrição de bloqueadores dos receptores da angiotensina (BRA), bloqueadores dos canais de cálcio e estatinas aumentou significativamente, enquanto a de digoxina, betabloqueadores não cardíacos e ácido acetilsalicílico (AAS) diminuiu na primeira teleconsulta. A maioria dos exames complementares solicitados era de baixa complexidade e custo: eletrocardiograma (28,2%), radiografia de tórax (14%), ecocardiograma (64,5%) e exames de sangue (71,8%). Para 2,1% dos pacientes, foram indicadas intervenções, e 8% recebeu alta após a primeira consulta.

**Conclusão:**

A teleconsulta cardiológica sob demanda contribui para a otimização do tratamento das doenças cardíacas. A maioria dos pacientes foi encaminhada com diagnósticos sindrômicos sem exames complementares prévios. A avaliação especializada solicitada geralmente estava disponível localmente e com baixo custo, mas impedia a alta precoce. Capacitação local poderia otimizar o encaminhamento.

## Introdução

A telemedicina (TM) se tornou um recurso essencial para o sistema de saúde, pois fornece assistência com boa relação custo-benefício por meio de ações imediatas.^1^ Embora diversos tipos de avaliações virtuais de pacientes estejam em prática desde a década de 1970, a avaliação por TM aumentou na década atual.^2^ Evidências científicas progressivas corroboram o uso da TM em diversos cenários.^3^ Algumas populações, principalmente em áreas remotas com barreiras geográficas, carecem cronicamente de assistência médica presencial e financiamento.^4^ A TM tem imenso potencial de fornecer avaliações essenciais a essas populações, seja por um generalista ou especialista.^5 , 6^

**Figura 1:**
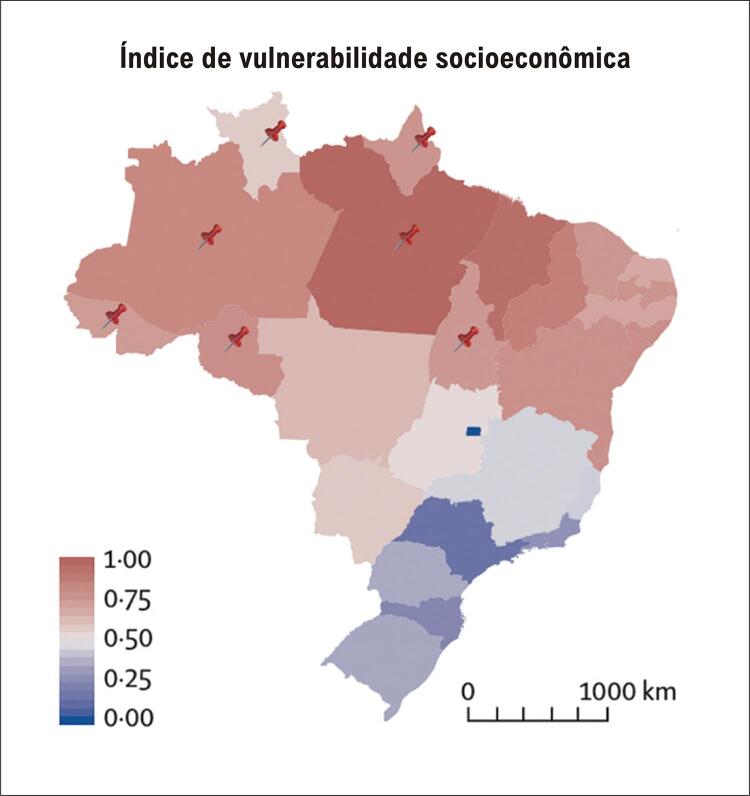
– Mapa do Brasil representando os valores dos índices de vulnerabilidade socioeconômica. Os estados marcados com alfinete vermelho correspondem à Região Norte do país. Adaptado de Rocha et al.8

Segundo o último Censo do Instituto Brasileiro de Geografia e Estatística, a Região Norte do Brasil possui mais de 12.500.000 habitantes, sendo que pelo menos 20% vivem em áreas remotas,^7^ distantes dos centros urbanos ou distantes de locais habitados, e de difícil acesso. Essa região é a maior do Brasil, caracterizada pela floresta amazônica (densa, com dificuldade de acesso a serviços de saúde), com a menor densidade populacional e Índice de Desenvolvimento Humano do país ( Figura 1 ).^8^ Além disso, a Região Norte tem a menor densidade de serviços médicos do país, com uma média de um médico por mil habitantes, mas chegando a 0,2 por mil habitantes em áreas remotas.^9^ Em uma análise recente das estatísticas de saúde no Brasil, as doenças cardiovasculares foram a principal causa de morte nessa região, com pior prognóstico do que em outras áreas com maiores níveis de desenvolvimento.^10^ Em fevereiro de 2020, o governo brasileiro lançou um programa de teleconsulta em cardiologia nesta região, com o objetivo de reduzir a carga de doenças cardiovasculares.^11^

As evidências corroboram o estabelecimento da TM como estratégia para altos níveis de satisfação relacionados às avaliações, bem como redução dos tempos gastos por especialistas e morbimortalidade em alguns grupos de doenças cardiovasculares (principalmente pacientes com insuficiência cardíaca).^12 , 13^ No entanto, os principais motivos de encaminhamento para especialistas, características clínicas, status de tratamento atual e tratamento por TM não são totalmente conhecidos. Esses dados podem presumivelmente realinhar e adaptar o projeto atual e orientar outras iniciativas remotas com foco em uma estratégia custo-eficiente.

O objetivo deste estudo foi analisar os dados de atendimento relacionados às teleconsultas cardiológicas por parte de pacientes da região brasileira com as cidades mais isoladas.

## Materiais e métodos

Este foi um estudo descritivo retrospectivo envolvendo um único centro de TM (Hospital Israelita Albert Einstein) que foi referência para 104 centros com atendimento presencial na Região Norte do Brasil relacionados ao programa de assistência médica especializada do Programa de Apoio ao Desenvolvimento Institucional do Sistema Único de Saúde (PROADI) por meio de TM, do Ministério da Saúde, Brasil ( Figura Central ).

**Figura Central:**
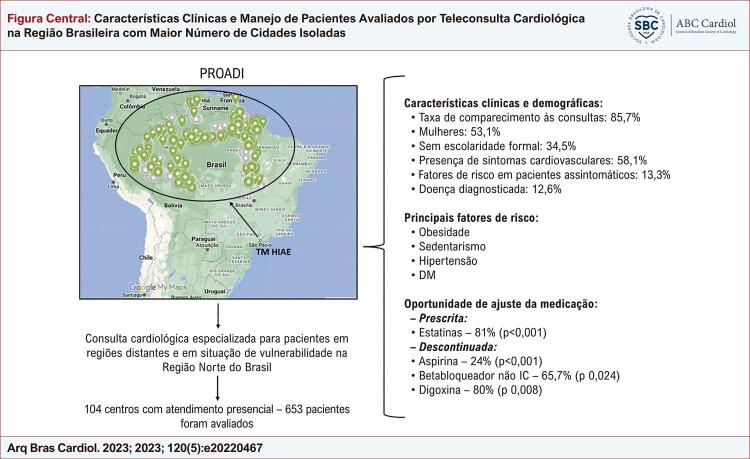
: Características Clínicas e Manejo de Pacientes Avaliados por Teleconsulta Cardiológica na Região Brasileira com Maior Número de Cidades Isoladas

Benefícios da teleconsulta cardiológica em cidades isoladas. O mapa ilustra as unidades de saúde da Região Norte do Brasil integrantes do programa de atendimento médico especializado por telemedicina. As marcações em verde indicam os locais dos serviços participantes. As marcações em cinza indicam os locais para futuras instalações de equipamentos. Os asteriscos indicam locais extras não previstos inicialmente pelo Ministério da Saúde. Mapa do Google Maps, idioma português. DM: diabetes melito; PROADI: Programa de Apoio ao Desenvolvimento Institucional do Sistema Único de Saúde por meio de TM, do Ministério da Saúde.

**Figura 2:**
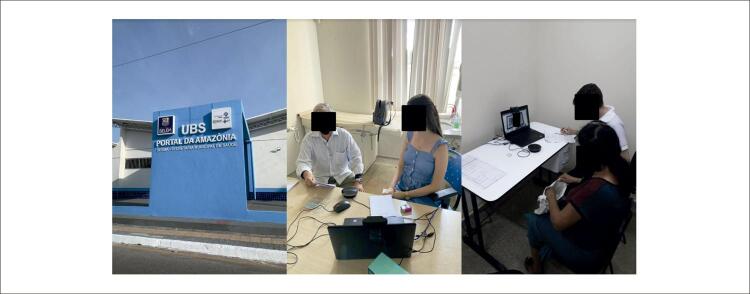
– Imagem externa de uma unidade de saúde. Consulta cardiológica sob demanda.

Os pacientes foram previamente avaliados por médicos generalistas da comunidade que solicitaram consulta especializada em cardiologia. De acordo com os procedimentos de agendamento do sistema de saúde local, os pacientes foram encaminhados para consulta por TM como alternativa à consulta com especialista. Todas as avaliações remotas deste programa incluíram o paciente ao lado do clínico geral da unidade de saúde com o cardiologista do centro de TM em tempo real. Todas as instalações contavam com equipamentos modernos para uma conexão rápida à internet com áudio e vídeo. Realizou-se a teleconsulta após a confirmação do perfeito funcionamento de áudio e imagem ( Figura 2 ). Os médicos de plantão que trabalhavam no posto de saúde não eram necessariamente os médicos que encaminhavam o paciente para avaliação especializada. Tiveram acesso ao prontuário do paciente e ao motivo do encaminhamento e participaram conjuntamente da teleconsulta. Os pacientes tiveram consultas com duração de 30 minutos, e todos os dezoito cardiologistas da telemedicina trabalharam por quatro horas consecutivas e foram previamente treinados de acordo com os protocolos institucionais de TM. Ao final de cada avaliação, o clínico geral do local recebia o laudo online do especialista e prosseguia à avaliação com reforço de esclarecimentos, agendamento de exames e prescrições, além do preenchimento do prontuário local.

Incluímos pacientes consecutivos avaliados de fevereiro de 2020 (início do programa) a outubro de 2021. O atendimento foi substancialmente interrompido ao longo desta operação devido à pandemia de COVID-19, com retomada progressiva desde março de 2021. Do prontuário da TM, foram analisados os seguintes parâmetros: motivo do encaminhamento, dados demográficos, histórico clínico e exame físico, bem como avaliação pré e pós-TM quanto a exames, medicamentos, diagnósticos e prescrições.

### Análise estatística

As principais estatísticas foram predominantemente descritivas. Utilizou-se o teste de Kolmogorov-Smirnov para a verificação de normalidade dos dados. As variáveis contínuas foram descritas como média e desvio padrão (DP), e as variáveis categóricas foram descritas como números absolutos e porcentagens. A única comparação foi realizada entre as prescrições por meio do teste de McNemar. Valores com p<0,05 foram considerados estatisticamente significativos, e utilizou-se o software IBM-SPSS para Windows versão 22.0 para cálculos estatísticos.

## Resultados

Foram avaliados 653 pacientes agendados de 17/02/2020 a 04/10/2021, com taxa de comparecimento às consultas de 85,7%. A maioria era do sexo feminino, com idade média de 54,2 anos. Houve forte evidência de pacientes de baixa renda caracterizados por nível educacional; o principal motivo do encaminhamento foi a presença de pelo menos um dos seguintes sintomas: dispneia, dor torácica, síncope ou palpitações. A maioria dos pacientes não apresentou eventos cardiovasculares maiores, apesar da presença de fatores de risco para doenças cardiovasculares na maioria dos pacientes avaliados; 60,1% apresentava hipertensão, seguida de dislipidemia, tabagismo e diabetes mellitus. Poucos referiram praticar atividade física regular ( Tabela 1 ). Apenas alguns sintomas relacionados a doenças cardiovasculares foram explorados durante a teleconsulta, e um médico local guiado por um cardiologista remoto realizou um exame físico. O sintoma mais comumente relatado foi dor precordial, mas apenas 26,1% dos pacientes tinha alta suspeita de cardiopatia isquêmica. Houve relatos de dispneia por 1/3 dos pacientes, e alguns deles se encontravam na classe funcional III ou IV da NYHA. Surpreendentemente, 151 (26,8%) pacientes palpitações, enquanto a síncope foi raramente observada. Mediu-se frequência cardíaca e pressão arterial em todos os pacientes antes da teleconsulta, com média e desvio padrão de 76,9±13,8, 136,4±67,3 (sistólica) e 82,1±13,9 (diastólica), respectivamente. Muitos pacientes apresentaram exame físico normal, com poucos casos de sopro ou sinais de insuficiência cardíaca detectados ( Tabela 2 ).

**Tabela 1:** – Comparecimento e características demográficas e clínicas basais

Variável	Descrição
	**média±DP**
Idade (anos)	54,2±16,5
	**n (%)**
Comparecimento positivo	563 (85,7)
**Sexo**	
Masculino	264 (46,9)
Feminino	299 (53,1)
**Nível de Escolaridade**	
Nenhuma escolaridade formal	181 (34,5)
Ensino fundamental completo	56 (10,7)
Ensino fundamental completo	68 (13)
Ensino médio incompleto	53 (10,1)
Ensino médio completo	135 (25,8)
Nível universitário incompleto	15 (2,9)
Nível universitário completo	16 (3,1)
**Motivo do encaminhamento**	
Pelo menos um destes: dispneia/dor torácica/síncope/edema/palpitação	327 (58,1)
Fatores de risco para doenças cardiovasculares	75 (13,3)
Arritmia diagnosticada	31 (5,5)
Doença arterial coronariana diagnosticada	18 (3,2)
Insuficiência cardíaca diagnosticada	13 (2,3)
Valvopatia diagnosticada	9 (1,6)
Outras	90 (16)
**Histórico clínico basal**	
Hospitalização cardiovascular anterior	52 (9,2)
Angioplastia coronariana	11 (2)
Revascularização miocárdica	11 (2)
Acidente vascular cerebral	28 (5)
Doença arterial obstrutiva periférica	7 (1,2)
Hipertensão	338 (60,1)
Glicemia de jejum alterada	28 (5)
Diabetes mellitus	87 (15,5)
Dislipidemia	164 (29,1)
Atividade física regular	53 (9,4)
Tabagismo	103 (18,3)
**Condição crônica não cardiovascular**	
Doenças respiratórias	18 (3,2)
Doenças do aparelho digestivo	24 (4,3)
Doenças do aparelho geniturinário	21 (3,7)
Doenças reumatológicas	20 (3,6)
Doenças psiquiátricas/neurológicas	41 (7,3)
Doenças endocrinológicas	35 (6,2)
Doenças hematológicas/imunes	4 (0,7)
Alcoolismo	24 (4,3)

**Tabela 2:** – Características clínicas observadas na consulta e exames complementares recentes

Variável	Descrição
	**n (%)**
Dispneia	167 (29,7)
**Classe Funcional da NYHA**	
I/II	142 (95,5)
III/IV	25 (4,5)
Dor precordial	277 (49,2)
**Tipos de dor precordial**	
Tipo D — definitivamente não é angina	96 (34,6)
Tipo C — provavelmente não é angina	109 (39,3)
Tipo B — provavelmente é angina	44 (15,9)
Tipo A — definitivamente é angina	28 (10,2)
**Angina grau no tipo A/B segundo a Canadian Cardiovascular Society**	
I/II	55 (76,6)
III/IV	17 (23,4)
Palpitações	151 (26,8)
Síncope	14 (2,5)
Sopro	26 (4,6)
Estase jugular	8 (1,4)
Edema periférico	32 (5,7)
Estertores na ausculta dos pulmões	13 (2,3)
**Eletrocardiograma**	
normal, n (%)	252 (59,3)
fibrilação/flutter atrial, n (%)	11 (2,6)
qualquer grau de síndrome do nó sinusal ou bloqueio atrioventricular	18 (4,2)
qualquer sobrecarga de câmaras cardíacas	69 (16,2)
repolarização alterada inespecífica	38 (8,9)
outros	37 (8,7)
Radiografia de tórax sugestiva de qualquer doença cardíaca	21 (45,7) / n=46
**Ecocardiograma (n=82)**	
normal	39 (47,6)
fração de ejeção ventricular esquerda <50%	7 (8,5)
hipertrofia ventricular esquerda	18 (22)
valvopatia importante	12 (14,6)
Hipocinesia segmentar ventricular esquerda	5 (6,1)
outros	1 (1,2)
Holter alterado (batimentos ventriculares prematuros, taquicardia, bradicardia) (n=30)	16 (53,3)
Teste ergométrico com alterações de isquemia (n=20)	0 (0)
**Cateterismo coronariano cardíaco (n=20)**	
normal	7 (35)
Doença arterial coronariana não significativa	5 (25)
Doença arterial coronariana obstrutiva	8 (40)
	**média****+****DP**
Frequência cardíaca, média + DP	76,9 ± 13,8
Pressão arterial sistólica, média + DP	136,4 ± 67,3
Pressão arterial diastólica, média + DP	82,1 ± 13,9

Foram realizados eletrocardiogramas em todos os pacientes, sendo a maioria normal. Outros exames, como ecocardiogramas e testes ergométricos, foram realizados em menor número de pacientes, também com resultados primordialmente normais. Os casos foram conduzidos comparando-se prescrições basais anteriores com prescrições de teleconsulta e descrevendo-se os exames complementares necessários ( Tabela 3 ). Todas as prescrições de medicamentos foram alteradas em alguma medida, mas apenas ARAs, digoxina, betabloqueadores não-IC, AAS, bloqueadores dos canais de cálcio e estatinas foram estatisticamente significativos. A maioria dos novos exames solicitados era de baixa complexidade e baixo custo, como eletrocardiograma, radiografia de tórax, ecocardiograma e exames de sangue. O teste ergométrico foi a avaliação não invasiva mais comum da cardiopatia isquêmica, sendo solicitado em 31,8% dos casos. Exames mais complexos, como tomografia computadorizada (TC) e cateterismo cardíaco, eram incomuns. Muito poucos pacientes foram indicados para intervenção cirúrgica ou transcateter.

**Tabela 3:** – Administração de medicamentos e novos exames solicitados

Medicação (n=563)	Prévia n (%)	Após a teleconsulta n (%)	p*
IECA	31 (5,5)	47 (8,3)	0,596
BRA II	94 (16,7)	123 (21,8)	<0,001
Betabloqueador para IC	26 (4,6)	39 (6,9)	0,461
Espironolactona	19 (3,4)	13 (2,3)	0,201
Sacubritril – valsartana	0 (0)	1 (0,2)	>0,999
Diuréticos	62 (11)	78 (13,9)	0,579
Digoxina	8 (1,4)	2 (0,4)	0,008
Betabloqueador não-IC	50 (8,9)	26 (4,6)	0,024
AAS	75 (13,3)	57 (10,1)	<0,001
Inibidores de P2Y12	5 (0,9)	7 (1,2)	>0,999
Varfarina	2 (0,4)	2 (0,4)	0,289
DOAC	1 (0,2)	5 (0,9)	0,375
Bloqueador de canal de cálcio	33 (5,9)	36 (6,4)	0,007
Antidiabéticos orais	2 (0,4)	1 (0,2)	0,289
Insulina	5 (0,9)	3 (0,5)	>0,999
Novos antidiabéticos orais	2 (0,4)	1 (0,2)	0,289
Estatina	44 (7,8)	80 (14,2)	<0,001
Fibrato	5 (0,9)	1 (0,2)	0,727
Nitrato	7 (1,2)	4 (0,7	0,804
**Exames solicitados, indicação de intervenção e alta (n=563)**		**n (%)**	
Ecocardiograma		159 (28,2)	
Radiografia de tórax		83 (14,7)	
Ecocardiograma		363 (64,5)	
Exames de sangue		404 (71,8)	
Teste ergométrico/cintilografia miocárdica		179 (31,8)	
MAPA		86 (15,3)	
Qualquer ultrassonografia		18 (3,2)	
Qualquer TC		36 (6,4)	
Cateterismo cardíaco		31 (5,5)	
Intervenção		12 (2,1)	
Alta		45 (8)	

IECA: inibidores da enzima conversora de angiotensina; BRA: bloqueadores dos receptores da angiotensina; IC: insuficiência cardíaca; AAS: ácido acetilsalicílico; DOAC: anticoagulantes orais diretos; MAPA: Monitorização ambulatorial da pressão arterial; TC: tomografia computadorizada. *Teste de McNemar.

## Discussão

Este é o primeiro estudo a analisar as características e conduta das consultas cardiológicas por TM sob demanda para populações de baixa renda em áreas remotas do Brasil. Considerando a nova implantação desse programa e as dificuldades relacionadas às características geográficas e demográficas da Região Norte do Brasil, que possui áreas isoladas e baixa proporção de médicos por habitante, a taxa de atendimento positivo de 85,7% foi satisfatória. Por exemplo, os médicos locais relataram que muitos pacientes tiveram dificuldades para acessar o local de consulta, com viagens de barco demorando mais de um dia. Além disso, muitos pacientes precisavam de acompanhantes para ajudá-los a entender as explicações básicas sobre o tratamento.

Percebemos algumas diferenças nas estatísticas mais atuais ao analisar os resultados da coleta de dados. Por exemplo, o número de teleconsultas cardiológicas para pacientes do sexo feminino foi ligeiramente superior ao do sexo masculino (53,1 vs. 46,9%), diferindo dos resultados do artigo de estatísticas cardiovasculares brasileiras de 2020, em que a prevalência global de doenças cardiovasculares (DCV) na Região Norte foi maior no sexo masculino (54%).^10^

Os principais motivos de encaminhamento foram dor torácica, dispneia, palpitações e síncope. Em geral, os perfis de complexidade dos pacientes avaliados foram considerados baixos. Foram observadas características clínicas de baixa probabilidade de angina e doença arterial coronariana (resultando no baixo número de intervenções diagnósticas e terapêuticas indicadas). A maioria dos pacientes com dispneia foi classificada como dispneia não limitante, com características multifatoriais. Observou-se também baixa taxa de infartos prévios, cirurgia de revascularização do miocárdio e disfunção ventricular esquerda. A incidência de síncope foi baixa, e a anormalidade mais significativa identificada no teste de Holter de 24 horas foram extrassístoles isoladas.

Na avaliação clínica, os principais fatores de risco identificados para doenças cardiovasculares foram obesidade, sedentarismo, hipertensão e diabetes mellitus, condizente com o aumento mundial da prevalência da síndrome metabólica, conforme demonstrado por Rissardo et al., que estudaram os perfis de risco cardiovascular de homens e mulheres em Santa Maria, pequena cidade da região sul do país, no período de 2012 a 2016.^14^ Curiosamente, a prevalência de fumantes foi baixa, talvez pela eficácia das campanhas de conscientização contra o tabagismo.

Os níveis médios de pressão arterial sistólica e diastólica apresentaram tendência à pré-hipertensão. No entanto, possíveis vieses de medição e hipertensão do avental branco devem ser considerados.^15^

Também observamos que as teleconsultas cardiológicas foram uma excelente oportunidade para ajustes de medicamentos. Quando comparamos os dados do estudo REACT 2013, que avaliou os perfis de prescrição de medicamentos de acordo com as evidências, observamos que a taxa de inibidores da enzima conversora de angiotensina (IECA) prescritos (53%) foi semelhante à de nossos achados.^16^ Antes das teleconsultas, 13% dos pacientes faziam uso de antiplaquetários (somente AAS), número bem inferior ao avaliado no estudo REACT (78%); no entanto, após as teleconsultas, o número diminuiu ainda mais significativamente.^16^ A grande maioria dos usuários de AAS o fazia desnecessariamente, pois a taxa de eventos cardiovasculares prévios era baixa. Talvez a mudança mais significativa tenha ocorrido nas prescrições de estatinas, que praticamente dobraram após as teleconsultas. Houve pouco uso de estatinas (7,8%) antes das teleconsultas, mesmo entre os pacientes com indicação de prevenção primária.

Esse achado é consistente com o perfil de risco esperado para uma população com fatores de risco não controlados. Nascimento et al.^17^ analisaram a prevalência do uso de estatinas no Brasil; dos 6.511 pacientes entrevistados, apenas 9,4% faziam uso de estatinas, sendo sinvastatina (90,3%), atorvastatina (4,7%) e rosuvastatina (1,9%) as mais utilizadas. A má adesão foi descrita por 6,5% dos pacientes.^17 - 19^ Apesar do aumento do número de pacientes em uso de estatinas após as teleconsultas, o número foi ainda inferior ao observado no REACT, talvez pelo maior número de pacientes com maior risco cardiovascular.^16^ O uso de betabloqueador fora do contexto de insuficiência cardíaca ou insuficiência coronariana foi suspenso em 65,7% dos pacientes, principalmente para tratamento de hipertensão arterial isolada (sem outras comorbidades). Os betabloqueadores representam a terapia de segunda linha no tratamento dessa patologia.^15^ Digitalis foi descontinuado em 80% dos pacientes, pois as diretrizes atualizadas de insuficiência cardíaca recomendam a digoxina em algumas situações.^20 , 21^ Houve grande dificuldade econômica para as prescrições farmacológicas mais atuais, como anticoagulantes orais diretos (DOACs), sacubitril-valsartan e antidiabéticos mais recentes, dado o alto custo dos medicamentos para o perfil econômico da população e a indisponibilidade de programas governamentais de distribuição de medicamentos. Apenas um paciente estava em uso do composto sacubitril-valsartana antes da teleconsulta.

A baixa taxa de exames solicitados também se alinhou com a baixa complexidade das patologias. Quando necessário, foram priorizados exames mais simples e acessíveis, como eletrocardiograma e radiografia de tórax. Outra questão crítica foi a indisponibilidade local de alguns exames, como o ecocardiograma, obrigando a realização de ajustes e adaptações na prescrição médica. A análise desses achados sugere que a teleconsulta tem um excelente potencial para administrar os procedimentos mais atualizados e adequados para pacientes em locais remotos.^6 , 11 , 12^

Este estudo apresenta algumas limitações: houve apenas observação da prática, sem randomização ou comparação com pacientes atendidos presencialmente.

## Conclusão

A teleconsulta cardiológica sob demanda oferece a oportunidade de otimizar o tratamento médico de diversas cardiopatias. A maioria dos pacientes foi encaminhada com diagnósticos sindrômicos sem exames complementares prévios. A avaliação especializada solicitada geralmente estava disponível localmente e com baixo custo, mas impedia a alta precoce. O treinamento local presumivelmente poderia otimizar o fluxo de encaminhamento.
