# Supplementary material for: Características Clínicas e Manejo de Pacientes Avaliados por Teleconsulta Cardiológica na Região Brasileira com Maior Número de Cidades Isoladas
Source: Arq Bras Cardiol. 2023 Apr 27;120(5):e20220467. [Article in Portuguese] doi: 10.36660/abc.20220467 (PMC10263392; doi:10.36660/abc.20220467)
Supplement: Translation

# Translation: Clinical Features and Management of Patients Assessed by Cardiology Teleconsultation in the Brazilian Region with the Highest Number of Isolated Cities

**Authors:** Tarso Augusto Duenhas Accorsi, Renato Paladino Nemoto, Jairo Tavares Nunes, Antônio Fernando Barros de Azevedo, Flavio Tocci Moreira, Karen Francine Kohler, Karine de Amicis Lima, Bruna Dayanne Reges Amaral, Renata Albaladejo Morbeck, Carlos Henrique Sartorato Pedrotti

**Affiliations:** 1 Brazil Hospital Israelita Albert Einstein – Telemedicine, São Paulo, SP – Brazil

**Keywords:** Telecardiology, Remote Consultation, Telemedicine, Referral and Consultation, Suburban Population

## Abstract

**Background:**

Cardiovascular diseases are the leading cause of adult mortality. Geographically remote and low-income Brazilian regions lack specialized consultations. The telemedicine management of this population by cardiologists is not fully known.

**Objectives:**

To analyze cardiology teleconsultation in the Brazilian region with the highest number of isolated cities.

**Methods:**

From February 2020 to October 2021, patients from the North Region of Brazil evaluated by local general practitioners were referred for cardiological evaluation by telemedicine. Referral reasons, demographics, clinical history, physical examinations, tests, medications, and prescriptions pre- and post-telemedicine were analyzed (p<0.05 was considered statistically significant).

**Results:**

We analyzed 653 patients. The attendance rate was 85.7% (53.1% female, mean age: 54.2±6.5 years). The main reasons for referral were cardiovascular symptoms (58.1%) and risk factors among asymptomatic patients (13.3%). Only 12.6% had a diagnosed disease. Most patients had regular physical examinations and electrocardiograms. Few had recent complementary tests. The prescription of angiotensin receptor blockers (ARBs), calcium channel blockers and statins was significantly increased, while that of digoxin, noncardiac beta-blockers and acetylsalicylic acid (ASA) was decreased at the first teleconsultation. Most of the tests requested were of low complexity and cost: electrocardiogram (28.2%), chest X-ray (14%), echocardiogram (64.5%) and blood tests (71.8%). For 2.1% of patients, interventions were indicated, and 8% were discharged after the first consultation.

**Conclusion:**

On-demand cardiology teleconsultation contributes to heart disease treatment optimization. Most patients were referred with syndromic diagnoses without previous complementary tests. The specialist workup requested was usually available locally and at a low cost but precluded early discharge. Local training could optimize the referral.

## Introduction

Telemedicine (TM) has become an essential resource for the health system as it provides cost-effective care via prompt actions.^1^ Although multiple kinds of virtual patient assessments have been in practice since the 1970s, TM evaluation has increased in the current decade.^2^ Progressive scientific evidence supports the use of TM in diverse scenarios.^3^ Some populations, especially in remote areas with geographical barriers, chronically lack face-to-face healthcare and financing.^4^ TM has an incredible potential for delivering essential healthcare to these populations, whether by a generalist or specialist.^5 , 6^

According to the last Brazilian Institute of Geography and Statistics Census, the North Region of Brazil has over 12,500,000 inhabitants, and at least 20% live in remote areas^7^ that are distant from urban centers or far away from inhabited places and have difficult access. This region is the largest in Brazil, characterized by the Amazon forest (dense, with impaired access to healthcare facilities), with the country’s lowest population density and Human Development Index ( Figure 1 ).^8^ Additionally, the North Region has the lowest medical provider density in the country, with an average of one doctor per thousand inhabitants, but reaching 0.2 per thousand inhabitants in remote areas.^9^ In a recent analysis of health statistics in Brazil, cardiovascular diseases were the leading cause of death in this region, with a worse prognosis than in other areas with higher levels of development.^10^ In February 2020, the Brazilian government launched a program for teleconsultation in cardiology in this region, aiming to reduce the burden of cardiovascular diseases.^11^

**Figure 1:** – Map of Brazil representing socioeconomic vulnerability index values. States marked with a red pin correspond to the North Region of the country. Adapted from Rocha et al.8

Evidence supports the establishment of TM as a strategy for high levels of satisfaction related to assessments, as well as a reduction in specialist times and morbimortality in some cardiovascular disease groups (mainly heart failure patients).^12 , 13^ However, the main reasons for referral to specialists, clinical features, current treatment statuses, and management by TM are not entirely known. These data may presumably realign and adapt the current project and guide other remote initiatives in focusing on a cost-effective strategy.

The objective of this study was to analyze the attendance data of cardiology teleconsultation management of patients in the Brazilian region with the most isolated cities.

## Materials and methods

This was a retrospective descriptive study involving a single TM center (Hospital Israelita Albert Einstein) that was a reference for 104 in situ centers in the North Region of Brazil related to the specialized medical assistance program of the health system development program (PROADI, in the Portuguese acronym) through TM by the Ministry of Health, Brazil ( Central Illustration ).

**Central Illustration:** : Clinical Features and Management of Patients Assessed by Cardiology Teleconsultation in the Brazilian Region with the Highest Number of Isolated Cities

Benefits of cardiology teleconsultation in isolated cities. The map illustrates the health facilities in Brazil’s North Region that are members of the program of specialized medical assistance through telemedicine. Green markings indicate the locations of participating facilities, while gray markings indicate the locations for future equipment installation. Stars indicate extra locations not initially foreseen by the Ministry of Health. Map from Google Maps, Portuguese language. DM: diabetes mellitus; PROADI: specialized medical assistance program of the health system development program through TM by the Ministry of Health.

Patients were previously assessed by community general practitioners who requested specialist cardiology consultation. According to local health system booking procedures, patients were referred for TM consultation as an alternative to the specialist encounter. All remote assessments of this program included the patient alongside the health facility general practitioner with the cardiologist from the TM center in real time. All facilities were equipped with modern devices allowing a fast internet connection with audio and video. Teleconsultation was performed after confirming perfect audio and image function ( Figure 2 ). On-site physicians who worked in the health center were not necessarily the physicians who referred the patient for specialized evaluation. They had access to the patient’s medical records and the reason for the referral, and jointly participated in the teleconsultation. Patients had consultations lasting 30 minutes, and all eighteen telemedicine cardiologists worked for four consecutive hours and were previously trained according to institutional TM protocols. At the end of each evaluation, the on-site general practitioner received the online report from the specialist and continued evaluation with reinforcement of explanations, test scheduling, and prescriptions, in addition to filling out the local medical record.

**Figure 2:** – External image of a health facility. On-demand cardiology consultation.

We included consecutive patients evaluated from February 2020 (the beginning of the program) to October 2021. Care was substantially interrupted throughout this operation due to the COVID-19 pandemic, and there has been a progressive resumption since March 2021. From TM medical records, the following parameters were analyzed: reason for referral, demographic data, clinical history, and physical examination, as well as pre- and post-TM evaluation regarding tests, medications, diagnoses, and prescriptions.

### Statistical analysis

The main statistics were predominantly descriptive. The Kolmogorov‒Smirnov test was used to confirm data normality. Continuous variables were described as mean and standard deviation (SD), and categorical variables were described as absolute numbers and percentages. The only comparison was conducted between prescriptions using McNemar’s test. Values with p<0.05 were considered statistically significant, and we used IBM-SPSS for Windows version 22.0 software for statistical calculations.

## Results

A total of 653 patients scheduled from 02/17/2020 to 10/04/2021 were evaluated, with a consultation attendance rate of 85.7%. The majority were female, with a mean age of 54.2 years. There was strong evidence of low-income patients characterized by educational level; the main reason for referral was the presence of at least one of the following symptoms: dyspnea, chest pain, syncope, or palpitations. Most of the patients had not had any major cardiovascular events, despite the presence of risk factors for cardiovascular diseases among most of the evaluated patients; 60.1% had hypertension, followed by dyslipidemia, smoking and diabetes mellitus. Few reported regular physical activity ( Table 1 ). Only some cardiovascular-related symptoms were explored during teleconsultation, and a local physician guided by a remote cardiologist performed a physical examination. The most commonly reported symptom was chest pain, but only 26.1% of the patients were highly suspected to have ischemic heart disease. Dyspnea was reported in 1/3 of the patients, and a few of them were in NYHA functional class III or IV. Surprisingly, palpitations were noticed in 151 (26.8%) patients, while syncope was uncommonly observed. Heart rate and blood pressure were measured for all patients before teleconsultation and had a mean and standard deviation of 76.9±13.8, 136.4±67.3 (systolic), and 82.1±13.9 (diastolic), respectively. Many patients had a normal physical examination with few cases of murmur or signs of heart failure detected ( Table 2 ).

**Table 1:** – Attendance and baseline demographic and clinical features

Variable	Description
	**mean ± SD**
Age (Years)	54.2±16.5
	**n (%)**
Positive attendance	563 (85.7)
**Gender**	
Male	264 (46.9)
Female	299 (53.1)
**Educational level**	
No formal education	181 (34.5)
Elementary incomplete	56 (10.7)
Elementary complete	68 (13)
Incomplete high school	53 (10.1)
Complete high school	135 (25.8)
Incomplete university	15 (2.9)
Complete university	16 (3.1)
**Reason for referral**	
At least one: dyspnea/chest pain/syncope/edema/palpitation	327 (58.1)
Risk factors for cardiovascular disease	75 (13.3)
Diagnosed arrhythmia	31 (5.5)
Diagnosed coronary artery disease	18 (3.2)
Diagnosed heart failure	13 (2.3)
Diagnosed valvular heart disease	9 (1.6)
Others	90 (16)
**Baseline medical history**	
Previous cardiovascular hospitalization	52 (9.2)
Coronary angioplasty	11 (2)
Coronary artery bypass graft	11 (2)
Stroke	28 (5)
Peripheral obstructive artery disease	7 (1.2)
Hypertension	338 (60.1)
Altered fasting blood glucose	28 (5)
Diabetes mellitus	87 (15.5)
Dyslipidemia	164 (29.1)
Regular physical activity	53 (9.4)
Smoking	103 (18.3)
**Non-cardiovascular chronic condition**	
Respiratory diseases	18 (3.2)
Digestive system diseases	24 (4.3)
Genitourinary system diseases	21 (3.7)
Rheumatologic diseases	20 (3.6)
Psychiatric/neurological diseases	41 (7.3)
Endocrinological diseases	35 (6.2)
Hematologic/immune diseases	4 (0.7)
Alcoholism	24 (4.3)

**Table 2:** – Consultation clinical features and recent complementary tests

Variable	Description
	**n (%)**
Dyspnea	167 (29.7)
**NYHA functional class**	
I/II	142 (95.5)
III/IV	25 (4.5)
Chest Pain	277 (49.2)
**Types of chest pain**	
Type D - definitely not angina	96 (34.6)
Type C - probably not angina	109 (39.3)
Type B - probably angina	44 (15.9)
Type A - definitely angina	28 (10.2)
**Canadian Cardiovascular Society angina grade in type A/B**	
I/II	55 (76.6)
III/IV	17 (23.4)
Palpitations	151 (26.8)
Syncope	14 (2.5)
Murmur	26 (4.6)
Jugular stasis	8 (1.4)
Peripheral edema	32 (5.7)
Rales in lung auscultation	13 (2.3)
**Electrocardiogram**	
normal, n (%)	252 (59.3)
atrial fibrillation / flutter, n (%)	11 (2.6)
any degree of sick sinus syndrome or atrioventricular block	18 (4.2)
any chamber overload	69 (16.2)
unspecific altered repolarization	38 (8.9)
Others	37 (8.7)
Chest radiography suggested of any heart disease	21 (45.7) / n=46
**Echocardiogram (n=82)**	
Normal	39 (47.6)
left ventricular ejection fraction < 50%	7 (8.5)
left ventricular hypertrophy	18 (22)
severe valvular heart disease	12 (14.6)
segmental left ventricular hypokinesis	5 (6.1)
Others	1 (1.2)
Abnormal Holter (premature ventricular beats, tachycardia, bradycardia) (n=30)	16 (53.3)
Ischemic treadmill test (n=20)	0 (0)
**Cardiac coronary catheterization (n=20)**	
Normal	7 (35)
non-significant coronary artery disease	5 (25)
obstructive coronary artery disease	8 (40)
	**média****+****DP**
Heart rate, mean ± SD	76.9 ± 13.8
Systolic blood pressure, mean ± SD	136.4 ± 67.3
Diastolic blood pressure, mean ± SD	82.1 ± 13.9

Electrocardiograms were performed for all patients, and most were normal. Other tests, such as echocardiograms and exercise tests, were performed for fewer patients, also with primarily normal results. The cases were managed by comparing previous baseline prescriptions with teleconsultation prescriptions and describing the complementary tests required ( Table 3 ). All medicine prescriptions were changed to some degree, but only ARBs, digoxin, non-HF betablockers, ASA, calcium channel blockers, and statins were statistically significant. Most of the newly requested tests were of low complexity and low cost, such as electrocardiogram, chest radiography, echocardiogram and blood tests. The treadmill test was the most common noninvasive assessment of ischemic heart disease, requested in 31.8% of the cases. More complex tests, such as computed tomography (CT) scans and cardiac catheterization, were uncommon. Very few patients were indicated for surgical or transcatheter intervention.

**Table 3:** – Medicine management and new tests requested

Medication (n=563)	Previous n (%)	After teleconsultation n (%)	p*
ACEi	31 (5.5)	47 (8.3)	0.596
ARB II	94 (16.7)	123 (21.8)	<0.001
HF Beta blocker	26 (4.6)	39 (6.9)	0.461
Spironolactone	19 (3.4)	13 (2.3)	0.201
Sacubritril – valsartan	0 (0)	1 (0.2)	>0.999
Diuretic	62 (11)	78 (13.9)	0.579
Digoxin	8 (1.4)	2 (0.4)	0.008
Non-HF Beta blocker	50 (8.9)	26 (4.6)	0.024
ASA	75 (13.3)	57 (10.1)	<0.001
P2Y12 inhibitors	5 (0.9)	7 (1.2)	>0.999
Warfarin	2 (0.4)	2 (0.4)	0.289
DOAC	1 (0.2)	5 (0.9)	0.375
Calcium channel blocker	33 (5.9)	36 (6.4)	0.007
Oral antidiabetic	2 (0,4)	1 (0,2)	0.289
Insulin	5 (0,9)	3 (0,5)	>0.999
New oral antidiabetics	2 (0,4)	1 (0,2)	0.289
Statin	44 (7,8)	80 (14,2)	<0.001
Fibrate	5 (0,9)	1 (0,2)	0.727
Nitrate	7 (1,2)	4 (0,7	0.804
**Requested tests, intervention indication and discharge (n=563)**		**n (%)**	
Electrocardiogram		159 (28.2)	
Chest radiography		83 (14.7)	
Echocardiogram		363 (64.5)	
Blood tests		404 (71.8)	
Treadmill test/myocardial scintigraphy		179 (31.8)	
ABPM		86 (15.3)	
Any ultrasound		18 (3.2)	
Any CT scan		36 (6.4)	
Cardiac catheterization		31 (5.5)	
Intervention		12 (2.1)	
Discharge		45 (8)	

ACEi: angiotensin-converting enzyme inhibitors; ARB: angiotensin receptor blockers; ASA: acetylsalicylic acid; HF: heart failure; DOAC: direct-acting oral anticoagulants; ABPM: Ambulatory blood pressure monitoring; CT: computed tomography * McNemar test.

## Discussion

This is the first study to analyze the characteristics and management of on-demand TM cardiology consultations for low-income populations in remote areas of Brazil. Considering the new implementation of this program and the difficulties related to the geographic and demographic characteristics of the North Region of Brazil, which has isolated areas and a low proportion of physicians per inhabitant, the positive attendance rate of 85.7% was satisfactory. For example, local physicians reported that many patients had difficulties accessing the consultation location, with boat journeys taking more than one day. Additionally, many patients needed companions to help them understand basic explanations about the treatment.

We noticed some differences in the most current statistics when analyzing the data collection results. For example, the number of cardiological teleconsultations for female patients was slightly higher than that for males (53.1 vs. 46.9%), differing from the results of the 2020 Brazilian cardiovascular statistics article, in which the global prevalence of cardiovascular diseases (CVDs) in the North Region was higher among males (54%).^10^

The main reasons for referral were chest pain, dyspnea, palpitations, and syncope. In general, the complexity profiles of the evaluated patients were considered low. Clinical features of low probability of angina and coronary artery disease were observed (resulting in the low number of diagnostic and therapeutic interventions indicated). Most patients with dyspnea were classified as having nonlimiting dyspnea, with multifactorial characteristics. A low rate of previous infarctions, coronary artery bypass grafting, and left ventricular dysfunction was also observed. The incidence of syncope was low, and the most significant change identified on the 24-hour Holter test were isolated extrasystoles.

In clinical evaluation, the main risk factors identified for cardiovascular diseases were obesity, sedentary lifestyle, hypertension, and diabetes mellitus, consistent with the worldwide increase in the prevalence of metabolic syndrome, as demonstrated by Rissardo et al., who studied the cardiovascular risk profiles of men and women in Santa Maria, a small city in the southern region of the country, from 2012 to 2016.^14^ Interestingly, the prevalence of smokers was low, perhaps due to the effectiveness of campaigns raising awareness against smoking.

Mean systolic and diastolic blood pressure levels showed a tendency toward prehypertension. However, potential measurement biases and white coat hypertension must be considered.^15^

We also observed that cardiology teleconsultations were an excellent opportunity for medication adjustments. When we compared the REACT 2013 study data, which evaluated the drug prescription profiles according to the evidence, we noticed that the rate of angiotensin-converting enzyme (ACE) inhibitors prescribed (53%) was similar to that in our findings.^16^ Before teleconsultations, 13% of the patients were using antiplatelet drugs (ASA only), a number much lower than that evaluated in the REACT study (78%); however, after teleconsultations, the number decreased even more significantly.^16^ The vast majority of ASA users did so unnecessarily since the rate of previous cardiovascular events was low. Perhaps the most significant change was in statin prescriptions, which practically doubled after teleconsultations. There was little use of statins (7.8%) before the teleconsultations, even among patients with indications for primary prevention.

This finding is consistent with the expected risk profile for a population with uncontrolled risk factors. Nascimento et al.^17^ analyzed the prevalence of statin use in Brazil; of the 6,511 patients interviewed, only 9.4% used statins, with simvastatin (90.3%), atorvastatin (4.7%) and rosuvastatin (1.9%) being the most commonly used. Poor adherence was described by 6.5% of patients.^17 - 19^ Despite the increase in the number of patients using statins after teleconsultations, the number was still lower than that observed in REACT, perhaps due to the higher number of patients with increased cardiovascular risk.^16^ The use of betablockers outside the context of heart failure or coronary insufficiency was discontinued for 65.7% of patients, mainly for the treatment of isolated arterial hypertension (no other comorbidities). Betablockers represent second-line therapy in treating this pathology.^15^ Digitalis was discontinued for 80% of patients, as the updated heart failure guidelines recommend digoxin in some situations.^20 , 21^ There was great economic difficulty for the most current pharmacological prescriptions, such as direct-acting oral anticoagulants (DOACs)s, sacubitril-valsartan, and more recent antidiabetic drugs, given the high cost of medications for the population’s economic profile and the unavailability of government drug-dispensing programs. Only one patient was using the sacubitril-valsartan compound before the teleconsultation.

The low rate of tests requested also aligned with the low complexity of the pathologies. When necessary, priority was given to more straightforward and accessible tests, such as electrocardiograms and chest radiography. Another critical issue was the local unavailability of some tests, such as echocardiograms, which forced adjustments and adaptations to the medical prescription. The analysis of these findings suggests that teleconsultation has excellent potential for managing the most up-to-date and appropriate procedures for patients in remote locations.^6 , 11 , 12^

This study has some limitations: there was only observation of practice, without randomization or comparison with patients seen face-to-face.

## Conclusion

On-demand cardiology teleconsultation provides an opportunity to optimize the medical treatment of several heart diseases. Most patients were referred with syndromic diagnoses without previous complementary tests. The specialist workup requested was usually locally available and at low cost but precluded early discharge. Local training could presumably optimize the referral flow.
